# Auditory effects of autologous fat graft for TORP stabilization in the middle ear: a cadaveric study

**DOI:** 10.1186/s40463-018-0267-x

**Published:** 2018-02-17

**Authors:** Margaret Aron, Thomas G. Landry, Manohar Bance

**Affiliations:** 1 Division of Otolaryngology-Head and Neck Surgery, Université de Sherbrooke, Sherbrooke, QC Canada; 2 Division Otolaryngology-Head and Neck Surgery, Nova Scotia Health Authority, Dalhousie University, Halifax, NS Canada; 3 Division Otolaryngology-Head and Neck Surgery, Nova Scotia Health Authority, Dalhousie University, Halifax, NS Canada; 4 Centre Hospitalier Université de Sherbrooke, Service d’Otorhinolaryngoloie et Chirurgie Cervicofaciale, Site Hôtel-Dieu, 580 rue Bowen Sud, Sherbrooke, QC J1G 2E8 Canada

**Keywords:** TORP, Autologous fat graft, Middle ear prosthesis stabilization, Laser Doppler vibrometry

## Abstract

**Background:**

Total ossicular replacement prostheses (TORP) are often used to re-establish ossicular coupling of sound in an ear lacking a stapes supra-structure. The use of TORPs, however, is associated with a 2/3 five year failure rate due to their anatomic instability over time in the middle ear. The use of autologous fat to try and stabilize TORPs may improve long-term results with this challenging ossicular reconstruction technique.

**Methods:**

A cadaveric temporal bone model was developed and laser Doppler vibrometry was used to measure and record round window membrane vibration in response to sound stimulation under the following conditions: normal middle ear, middle ear filled with fat, normal middle ear with TORP prosthesis, TORP prosthesis with fat around its distal end and TORP prosthesis with fat filling the middle ear. Fourteen temporal bones were used.

**Results:**

There was a significant decrease in round window membrane velocity after filling the middle ear with fat in both the normal middle ear (− 8.6 dB; *p* < 0.0001) and prosthesis conditions (− 13.7 dB; *p* < 0.0001). However, there was no significant drop in round window membrane velocity associated with using fat around the distal end of the TORP prosthesis as compared to the prosthesis without fat condition (*p* > 0.05).

**Conclusions:**

Autologous fat around the distal end of a TORP prosthesis may not be associated with any additional hearing loss, as demonstrated in this cadaveric model. The additional hearing loss potentially caused by using fat to completely surround the prosthesis and fill the middle ear is probably not clinically acceptable at this time, especially given the unknown way in which the fat will atrophy over time in this context.

## Background

Re-establishing ossicular coupling of sound in an ear lacking a stapes supra-structure can be quite a challenge. Alloplastic prostheses, intended to be placed onto the intact footplate on one end and either the tympanic membrane (TM) or malleus handle on its other end, are often used to re-establish ossicular coupling in these cases. These prostheses, otherwise known as total ossicular replacement prostheses (TORP) have variable clinical results and have been disappointing in many cases. Despite being lightweight and biocompatible, these reconstructions tend to lack stability, with 2/3 of TORPs failing after 5 years [1] as demonstrated by a recurrent increase in the air bone gap on audiologic testing. Prosthesis displacement or tilting are the major causes of unsatisfactory hearing after this type of surgery [2–4]. Re-exploration of these failed reconstructions most often reveals a prosthesis having been displaced from its original position on the footplate. Factors such as recurrent middle ear fluid, tympanic membrane retraction, scar formation/fibrosis, and atelectasis of the tympanic membrane may displace a perfectly placed prosthesis. Unfortunately, many of these factors cannot be controlled [5]. As surgeons, we must try to maximize prosthesis stability to allow it to withstand these displacing forces.

Our experience with fat in the middle ear largely comes from its use in fat graft myringoplasty where it helps guide inflammatory cells to heal perforations. In this context, bulky fat grafts are dumb-belled through a tympanic membrane perforation. With healing, the bulkiness of the graft is lost, leaving a thickened and healed ear drum behind [6, 7]. We hypothesize that if we use fat to stabilize the TORP prosthesis intra-operatively it may offer stability during the healing period of the middle ear, helping resist displacement forces on the prosthesis and then may atrophy with time, leaving a ventilated middle ear space. In fact, the senior author (MB) uses this method clinically. The effect on hearing of using fat in the middle ear in this context, however, has not been studied. The objective of this study was to evaluate the effect on round window membrane vibration of autologous fat deposited into the middle ear under various conditions including that of using the fat to stabilize a TORP prosthesis.

## Methods

In order to evaluate round window membrane vibration a temporal bone model was designed on which we could use a laser Doppler vibrometer (LDV) to record and compare round window membrane (RWM) movement in response to acoustic tone stimulation in several conditions. The model and measuring technique are detailed below. Ethics review board approval was obtained from the Dalhousie Research Ethics Committee for this study.

### Temporal bone preparation

**Fig. 1:**
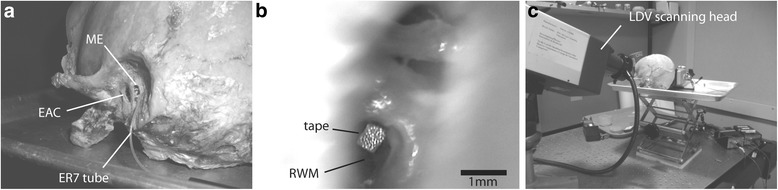
**a** Temporal bone with posterior tympanotomy to access middle ear. Custom holes drilled into the bony EAC for an ER-3A speaker tip (not shown) and an ER-7C microphone probe tube. **b** RWM exposed through the posterior tympanotomy with reflective tape on it. **c** Experiment setup in the soundproof booth with temporal bone and scanning head supported by a stability table to dampen floor vibrations

Fresh frozen cadaver temporal bones were used (*n* = 14) (Anatomy Gifts Registry, Hannover, USA) since inner ear fluid and soft tissue quality is preserved in these specimens. Soft tissue such as skin and muscle were removed from the lateral temporal bones and a canal wall up mastoidectomy was performed, as well as a posterior tympanotomy to expose the oval and round windows. The use of LDV to measure RWM movement requires a clear view of the entire RWM and thus the facial nerve was sacrificed as necessary to obtain optimal exposure of the RW. The round window niche was then drilled off as needed to have a direct view of the entire RWM. Finally, holes were drilled into the lateral bony external auditory canal (EAC) for an ER-3A speaker tip and an ER-7C microphone probe tube (Etymotic, Elk Grove Village, USA) (Fig. 1a). These were glued to the EAC wall for stabilization and the Er-7C microphone tip as ensured to be within 2 mm of the TM. The EAC was sealed by modeling clay during the experiments.

In order to obtain reliable LDV measurements, reflective polystyrene tape containing microbeads (3 M, Minneapolis, MN, USA) was placed on the RWM via the posterior tympanotomy to serve as a reflective target (Fig. 1b).

### Conditions under which RWM vibrations were recorded using laser Doppler vibrometry

Baseline (termed “Normal_NF”): Baseline LDV measurements of RWM movement in response to acoustic stimuli were measured with all native ossicles intact and the middle ear left ventilated.

Middle ear cavity filled with fat (termed “Normal_MEF”): we harvested fat from the temporalis fat pad and used it to fill the middle ear. Over-filling was avoided in order to prevent placing excessive pressure on the TM and ossicles. We ensured that fat surrounded all of the ossicles as well as the air containing middle ear space, including the epitympanum. Only the RWM was left uncovered and in view for LDV measurements. This condition was chosen to simulate the maximal dampening effect possible by replacing the entire middle ear air space with fat.

TORP without fat (termed “Pros_NF”): After disarticulating and removing the incus as well as the stapes suprastructure through the posterior tympanotomy, a titanium TORP prosthesis (TTP-VARIAC Total Prosthesis, Kurz, Dusslingen, Germany) was measured up using the sizing template and placed between the footplate and the posterosuperior TM.

TORP with fat around the footplate (termed “Pros_FPF”): the fat harvested from the temporalis fat pad was used to surround the prosthesis shaft on the stapes footplate. In all cases the distal bulged end of the TORP was completely covered by fat resulting in complete coverage of the oval window in all cases. This condition was chosen to predict the amount of dampening of sound one might expect when using fat to stabilize the distal end of the TORP on the footplate.

TORP with fat filling the middle ear (termed “Pros_MEF”): the conditions for “Normal_MEF” were repeated with the TORP in place.

### Laser Doppler vibrometer setup and recording software

A LDV (PSV-400 scanning head, OFV-5000 controller, Polytec PI, Tustin, CA, USA) and software (Polytec Scanning Vibrometer version 8.7) were used to measure and record movement of the RWM.

Settings of the software system:

- Average of 4 trials per point
- 640 ms recordings window with a sample frequency of 25.6 kHz
- Acoustic stimulation used was constant sine waves at the following frequencies: 250, 500, 1000, 2000, 3000, 4000, 6000, 8000 Hz
- Sound was presented in the EAC using the ER-3A speaker at 100 dB SPL (or to a maximum of 4 V input if 100 dB could not be attained). This intensity was confirmed with the ER-7C microphone.

Physical setup for LDV measurements: (Fig. 1c)

- The bone was placed in a soundproof booth.
- The temporal bone specimen and scanning head were supported by a stability table to dampen floor vibrations.
- Modeling clay was used to seal the lateral EAC and the holes around the ER-3A and ER-7C to provide an air-tight seal in order to attain the desired sound pressure levels within the EAC. Air-tight closure was confirmed when sound pressure emitted by the ER-3 into the EAC was measured to be at 100 dB SPL by the ER-7.
- The bone was oriented to have a direct full-on view on the RWM in order to most accurately measure its vibration velocity with the LDV.
- 5–7 points were arbitrarily placed over the entire area of reflective tape on the RWM.
- Where visibly possible (all conditions other than “fat filling ME” condition) 2–3 points were also chosen along the stapes or prosthesis, depending on the condition.
  - ◦ Setup did not provide the optimal angle for this measurement in every case but, when possible, measurement was completed for phase comparison with RWM movement. In all comparisons, the stapes and RWM were about 180° out of phase at low frequencies.
- The vibration velocity of the RWM and TORP/stapes, when possible, were measured by the laser in response to acoustic stimulation delivered through the EAC.

### Data analysis

RWM vibration velocities were normalized to sound pressure (m/s/Pa), converted to dB re:1 μm/s/Pa and analyzed using SPSS 23 software (IBM, Armonk, USA). A two-way repeated measures analysis of variance (ANOVA) was performed with Greenhouse-Geisser corrections for three different condition groupings: 1) Normal-NF vs Normal_MEF; 2) Normal_NF vs Pros_NF; and 3) Pros_NF vs pros_FPF vs Pros_MEF. The within-subject factors that were considered were condition and frequency, with post hoc contrasts for condition being simple type (reference of Normal_NF for tests 1 and 2, Pros_NF for test 3). The middle ear has a known frequency response curve. Therefore, any significant frequency main effects are not important results. However, significant condition × frequency interactions are more relevant effects, indicating frequency-specific differences between conditions. The three different ANOVAs were performed rather than just one containing all conditions in order to better interpret any potential frequency interaction effects and because different reference conditions were desired for examining different contrasts. This approach allowed us to only examine effects which were most relevant post hoc, avoiding having to perform 40 (5 conditions × 8 frequencies) comparisons, which would have increased the probability of a type I error. Type I error rate for the three ANOVAs was reduced to 0.05/3 = 0.0167.

## Results

**Fig. 2:**
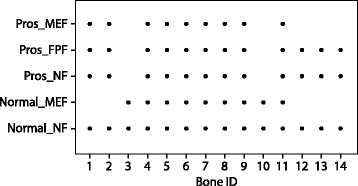
Number of temporal bones used for each experimental condition

Fourteen temporal bones were dissected. Because of technical issues during dissection (ex: TM perforation, accidental footplate removal with stapes suprastructure excision), some bones were unable to be used for certain conditions. Figure 2 details which bones had data available for each condition.

### Normal_NF vs Normal_MEF (*n* = 9) (Fig. 3)

The ANOVA showed that there was a significant decrease of 8.6 dB in RWM velocity when the middle was filled with fat (*F*(1,7) = 47.386, *p* < 0.0001). There was also a significant effect of frequency (*F*(2.281,15.965) = 23.355, *p* < 0.0001) on RWM velocity, but there was no significant condition × frequency interaction (*p* > 0.05).

**Fig. 3:**
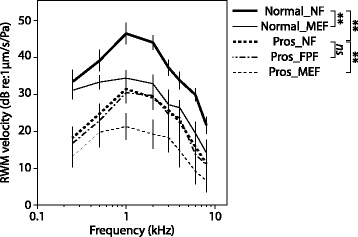
Round window membrane velocity as a function of frequency for all tested conditions. (ns = not significant. ** = statistically significant)

### Normal_NF vs Pros_NF (*n* = 12) (Fig. 3)

The ANOVA showed that there was a significant decrease of 13.7 dB in RWM velocity with the prosthesis in place compared to baseline (*F*(1,9) = 18.095, *p* = 0.002). There was also a significant frequency effect (*F*(1.476,13.285) = 9.914, *p* = 0.005) on RWM velocity, but no significant interaction (*p* > 0.05).

### Pros_NF vs Pros_FP fat vs Pros_MEF (*n* = 9) (Fig. 3)

The ANOVA showed that there was a significant effect of middle ear condition (*F*(1.904,13.329) = 23.405, *p* < 0.0001) and frequency (*F*(1.984,13.887) = 6.849, *p* = 0.009), but there was no significant interaction (*p* > 0.05). Contrasts showed there was no difference in RWM velocity with placement of fat only around the prosthesis footplate compared to the prosthesis alone (Pros_NF) (*p* > 0.05). There was, however, a significant 7.1 dB loss when fat was used to fill the entire middle ear with the prosthesis (Pros_MEF), as compared to the prosthesis with no fat (Pros_NF) (*p* < 0.0001).

## Discussion

Ossicular reconstruction in the absence of a stapes superstructure can be quite frustrating. Despite intra-operative satisfaction with prosthesis stability, long-term success rates (maintenance of air-bone gap < 20 dB) for TORP prosthesis have been estimated to be as low as 38% [1].

To improve TORP stability on the stapes footplate, and thus minimize its chances of displacing post-operatively, surgeons have tried many different techniques. The use of cartilage between the prosthesis head and TM has been described to not only reduce extrusion rates, but also to improve lateral stability of the prosthesis [8]. Efforts have also been made to stabilize the medial, shaft portion of the prosthesis using a fenestrated cartilage “shoe” placed onto the footplate within which the TORP would stand [9]. Fisch and May suggested placing a small spike at the end of the TORP shaft which would be placed into a fenestration in the footplate [10]. A titanium shoe has also been used to try and stabilize the medial end of the prosthesis onto the stapes footplate [11]. Recently, a “two-point stabilization” technique was described combining prosthesis head stabilization with cartilage combined with an areolar tissue graft between the prosthesis and footplate. Although this latter study demonstrated good post-operative audiologic outcomes, the mean follow-up was 8 months making conclusions about long-term stability difficult to draw [12].

Autologous fat could be a good choice of tissue to use for TORP stabilization given its biocompatibility and ease of harvest in otologic surgery. To better understand the use of fat to stabilize prosthesis position in the middle ear we conducted this study to evaluate the effect on sound transmission to the inner ear of putting variable amounts of fat in the middle ear and measuring RWM velocity in response to sound delivered to the tympanic membrane.

Firstly, our model seems to be appropriate given the 13.7 dB loss in RWM velocity when the virgin middle ear condition (Normal_NF) was compared to the condition with prosthesis alone (Pros_NF). In the literature, an air-bone gap of < 20 dB is generally considered a success for TORP prosthesis [13]. The 13.7 dB loss in our study falls into this range. The fact that the loss is a slightly less than that seen with clinical use of TORP probably reflects the fact that the rest of the middle ear in our cadaver study was completely normal, as opposed to the changes seen in chronic otitis media where TORP prostheses are most often used clinically.

In our study, adding fat around the footplate/distal end of the TORP, did not cause any significant additional hearing loss when compared to the unsupported TORP, and thus can presumably be used intra-operatively to stabilize the prosthesis without dampening sound transmission. However, clearly there are scarring effects and biological changes in the fat in real life that cannot be mimicked in a cadaveric study. We cannot easily predict what these would be, but given that even large amounts of fat in the middle ear have been used for fat myringoplasty [14], and that these must surround the ossicles by necessity, if the fat did indeed cause scar-related fixation of the ossicles we would expect a significant dampening effect on their vibration and an associated hearing loss in these cases. Clinical studies, however, show hearing results from these studies to be quite good [14], making it unlikely that fat has a severe long term effect on middle ear vibration after post-surgery biological changes.

Under the conditions of this study, however, filling the middle ear with fat both in intact middle ears and prosthesis conditions did seem to dampen sound transmission in a significant way, adding about another 8 dB loss. The dampening effect of fat filling the ear makes physiologic sense. Firstly, the fat pushing up against the TM mass loads the drum, similar to fluid in the middle ear [15]. Secondly, by reducing the middle ear air volume, the compliance of the middle ear is decreased, resulting in increased mechanical impedance to sound. As in the comparison of virgin middle ear (Normal_NF) vs prosthesis alone (Pros_NF), we may assume that the additional loss with middle ear fat in our model is likely somewhat underestimated given the absence of chronic middle ear changes in our model. Adding this hearing loss to the hearing loss already associated with having a TORP prosthesis is probably clinically unacceptable. But given the known tendency of fat grafts to atrophy significantly [16], we question whether the hearing loss associated with middle ear fat would be permanent or not. If the fat did atrophy in the middle ear over time, it could serve its potential advantage of TORP stabilization during the healing period. Once the prosthesis becomes fibrosed in place, the atrophy of the fat in the middle ear can then allow for more liberal vibration of the TM and ossicles making the actual reduction in hearing less than what has been measured in this study. Additional work would, however, be needed to evaluate how much fat atrophies as well as its pattern of atrophy in the middle ear and over what time frame to justify its use in this way.

## Conclusion

Fat is an easily available, biocompatible autologous graft material. Its use to stabilize the distal end of a TORP prosthesis on the footplate would not likely be associated with any additional hearing loss, as suggested by our cadaver model results. The additional hearing loss caused by using the fat to completely surround the prosthesis and abut the TM is probably not clinically acceptable at this time given the unknown way in which it will atrophy over time in this context.

## Abbreviations

EAC: External auditory canal
LDV: Laser Doppler vibrometry
RWM: Round window membrane
TM: Tympanic membrane
TORP: Total ossicular reconstruction prosthesis
